# Evaluation of exome variants using the Ion Proton Platform to sequence error-prone regions

**DOI:** 10.1371/journal.pone.0181304

**Published:** 2017-07-24

**Authors:** Heewon Seo, Yoomi Park, Byung Joo Min, Myung Eui Seo, Ju Han Kim

**Affiliations:** Seoul National University Biomedical Informatics (SNUBI), Div. of Biomedical Informatics, Seoul National University College of Medicine, Seoul, Korea; German Cancer Research Center (DKFZ), GERMANY

## Abstract

The Ion Proton sequencer from Thermo Fisher accurately determines sequence variants from target regions with a rapid turnaround time at a low cost. However, misleading variant-calling errors can occur. We performed a systematic evaluation and manual curation of read-level alignments for the 675 ultrarare variants reported by the Ion Proton sequencer from 27 whole-exome sequencing data but that are not present in either the 1000 Genomes Project and the Exome Aggregation Consortium. We classified positive variant calls into 393 highly likely false positives, 126 likely false positives, and 156 likely true positives, which comprised 58.2%, 18.7%, and 23.1% of the variants, respectively. We identified four distinct error patterns of variant calling that may be bioinformatically corrected when using different strategies: *simplicity region*, *SNV cluster*, *peripheral sequence read*, and *base inversion*. Local *de novo* assembly successfully corrected 201 (38.7%) of the 519 highly likely or likely false positives. We also demonstrate that the two sequencing kits from Thermo Fisher (the Ion PI Sequencing 200 kit V3 and the Ion PI Hi-Q kit) exhibit different error profiles across different error types. A refined calling algorithm with better polymerase may improve the performance of the Ion Proton sequencing platform.

## Introduction

Several studies have used the Ion Proton sequencing platform from Thermo Fisher to identify causal variants in many genetic disorders [1–3]. The Ion Proton sequencer can reportedly achieve a rapid turnaround at a low cost with high accuracy in the genotype calls of targeted genomic regions [4]. Despite its strengths, systematic errors are introduced in sequencing data due to the use of the polymerase reaction or mapper-calling algorithms, especially in homopolymer-rich and high-AT-content regions [5]. Understanding sequence data characteristics has become an essential prerequisite when attempting to discover genuine variants [6]. Reviewing every variant is also recommended for accurate analysis in medical genomics [7].

In the present study we investigated results of variant calling from 27 whole-exome sequencing (WES) data generated by the Ion Proton sequencer. We focused on 675 ultrarare variants that are not present in either the 1000 Genomes Project (T1GP; http://www.1000genomes.org/) [8] or the Exome Aggregation Consortium (ExAC) [9]. By inspecting the alignment statuses of sequence reads that affect variant calls, we first determined whether or not a variant call is a false positive. We then characterized error patterns and classified error cases into four distinct classes. In addition, we compared error-prone loci between two sequencing kits from Thermo Fisher (the Ion PI Sequencing 200 kit V3 [S200V3] and the Ion PI Hi-Q kit [HiQ]) in order to characterize their error profiles.

## Materials and methods

### Ethics statement

Eighteen and 9 of subjects were collected from Asan Medical Center and The Catholic University of Korea Seoul St. Mary’s Hospital, respectively. A research that involving human subjects and their genomic data was approved by Asan Medical Center Institutional Review Board (2014–1216) and The Catholic University of Korea Seoul St. Mary’s Hospital Institutional Review Board (KC15TIMI0055). Written informed consent was obtained from all subjects prior to participation.

### Data generation and sequencing

Genomic DNA extracted from peripheral blood cells was amplified into 175- to 250-bp DNA fragments to collect the protein coding regions of human genome DNA using the Ion Ampliseq Exome Panel (Thermo Fisher). Library construction was performed to load the DNA samples into the semiconductor chip using the Ion Ampliseq Exome Plus library kit covering 57,742,646 bp (1.85% of the human genome) as described in the manufacturer’s instructions (Thermo Fisher). The exon-enriched DNA libraries were sequenced using the Ion Proton platform following the manufacturer’s instructions (Thermo Fisher). Twenty-seven subjects were sequenced with S200V3 (*n* = 14) and HiQ (*n* = 13). The mean depth of exome sequencing ranged from 80× to 120×, which was sufficient for interrogating the exons for mutations. Single-nucleotide variants (SNVs) and short insertions/deletions (INDELs) were identified by a mapping alignment program from Thermo Fisher (version 4.4, Torrent Suite Software) with germ-line and low stringency settings. The cutoff values for the variant calling parameters were as follows: allele frequency, >0.1 (SNV) and >0.25 (INDEL); read quality, >15 (SNV) and >20 (INDEL); coverage, >5 (SNV) and >10 (INDEL); strand bias, <0.98 (SNV) and <0.9 (INDEL); relative read quality, >5 (SNV); common signal shift, <0.25; reference/variant signal shift, <0.2 (insertion); and reference/variant signal, shift <0.2 (deletions). Reads from the Ion Proton sequencer are heterogeneous in length, and we did not apply any additional filtering or trimming steps.

### Selecting error-prone loci

**Fig 1:**
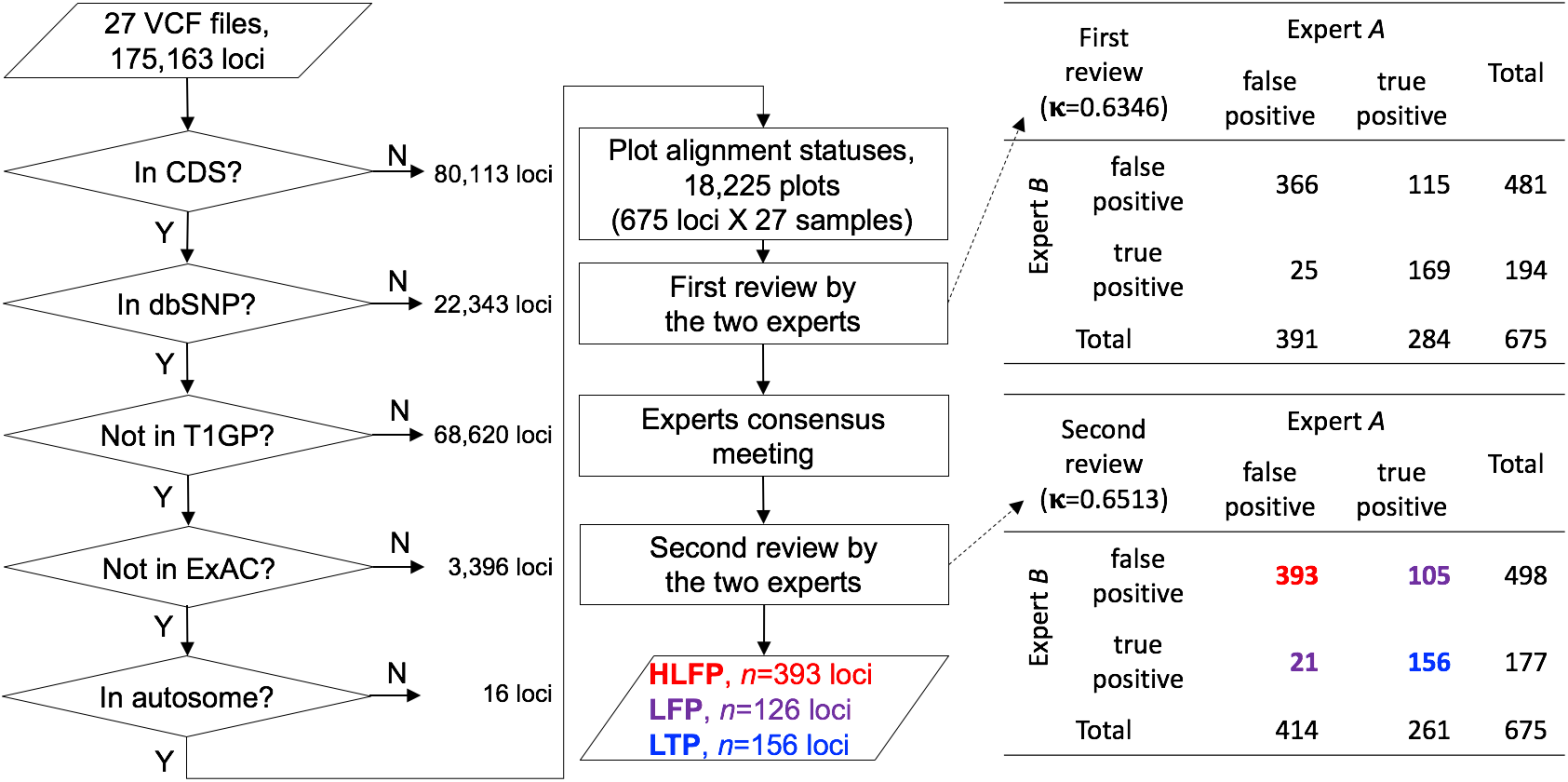
Workflow for selecting error-prone variants. This workflow shows the steps used to select the error-prone variants from 27 VCF (Variant Call Format) files of WES data. The number of loci excluded after each filtering step is indicated. The loci remaining after the filtering steps were classified into four distinct error types, which were considered to be false positives. Note that a locus could be classified into multiple categories if it satisfied multiple error-prone conditions. A call was a highly likely false positive (HLFP) when both experts agreed it was a false positive, a likely false positive (LFP) when the two experts disagreed, and a likely true positive (LTP) when both experts agreed it was a true positive call. Cohen’s kappa coefficient (κ) for the correlation between the two experts was 0.6513.

Error-prone variants were selected using multiple public databases (Fig 1). First, variants in the coding DNA sequence (CDS) were collected from version 75 of the Ensembl database (http://www.ensembl.org/) [10]. The intersection between CDS regions from the Ensembl and Ampliseq target regions was first measured. The Ion Ampliseq Exome Plus library kit captured 34.58% and 65.42% of noncoding and coding regions, respectively. Second, we extracted variants that have an rsID from build 142 of the dbSNP human database (http://www.ncbi.nlm.nih.gov/SNP/) [11]. Third, we excluded sequence variants appearing in phase 3 of T1GP [8] and ExAC [9]. These well-established human genome sequence variants were considered to be true positives based on the assumption that loci reported in highly curated databases are less likely to be errors. Fourth, we excluded variants in sex chromosomes. Finally, we created sequential image files capturing the alignment statuses of the remaining variants from the corresponding Binary Alignment/Map (BAM) files, with a single PNG file capturing a window size of 81 bp (40 bp either side of a single target variant). We additionally took snapshots of 201- and 501-bp windows when it was unclear whether a positive call was a true or false one. For the purpose of systematic and unbiased comparisons, alignment statuses for both positive and negative calls from all of the 27 BAM files were systematically captured into aligned image files. We developed an alignment viewer since existing genomic viewers such as the Integrative Genomics Viewer [12,13] are not capable of displaying insertion sequences with a full depth of sequence reads. Our new viewer was equipped with several enhanced features specifically designed to facilitate read alignment evaluations when guiding human inspections. Two bioinformatics experts manually inspected the read alignment status in every PNG file, and classified error-like alignment patterns into four error types. We assumed that all variant calls passed the given threshold during the Ion Proton variant calling process, focusing only on patterns of aligned reads that passed the quality control criteria in the variant calling process of the Torrent Suite Software. Descriptive statistics for each error type were computed based on the chromosomal position (loci) of the human reference genome (GRCh37).

### Calculating the weighted homopolymer rate

To evaluate the variants that are classified as a *simplicity region*, we computed the weighted homopolymer rate (WHR) proposed by the Broad Institute (https://www.broad.harvard.edu/crd/wiki/index.php/Homopolymer):

$$WHR=(\sum_{i=1}^{N}{n_{i}}^{2})/N$$ (1)

where *N* is the number of homopolymers in the sequence and *n*_*i*_ is the length of the *i*th homopolymer. A sequence is considered to be homopolymer-rich when its WHR is greater than 2.22. We performed simulations to calculate WHR for window sizes from 3 to 251 bp of the given loci, and decided that a size of 19 bp was optimal (9 bp either side of the identified variant position).

### Call error loci via local *de novo* assembly

We performed a variant-calling step using the HaplotypeCaller algorithm in the Genome Analysis Toolkit (GATK-HC), which discards the existing mapping information from Torrent Suite Software and applies a local *de novo* assembly algorithm to a given region [14]. We collected the start and end positions for each amplicon that harbored an HLFP or LFP call, and then applied the GATK-HC to a given amplicon region (from 156 to 240 bp) for each subject.

## Results

We applied WES to 27 individuals, which yielded 47,504±382 (mean±SD) and 49,504±599 SNVs and 2,150±118 and 1,968±202 INDELs with S200V3 (*n* = 14) and HiQ (*n* = 13), respectively (Table 1). Variants were identified at 175,163 loci in CDS regions (*n* = 95,050) and non-CDS regions (*n* = 80,113) in 27 individuals (Fig 1). First, we selected 95,050 loci present in the CDSs, resulting in 23,546±566 variants per sample being included in the CDSs. Second, we filtered out 22,343 variants without rsIDs from dbSNP 142 to exclude variants that have never been reported. Third, 68,620 loci reported in T1GP were excluded. Fourth, 3,396 loci found in ExAC were also excluded. Fifth, 16 loci that are located on sex chromosomes were excluded. This process yielded 675 loci that were manually investigated to determine the correctness of variant calls, and these ultrarare variants were evenly distributed in all of the samples (194.3±14.5, range = 163–224). There was no difference in the results obtained using the two kits for SNVs (Table 1). Finally, we created 18,225 PNG files (675 loci × 27 samples) with a full depth of sequence reads.

**Table 1:** Statistics of variants.

	SNVs per sample	*P*	INDELs per sample	*P*
**Whole exome (175,163 loci)**				
S200V3, *n* = 14	47,504.64±382.55	1.95E-10	2150.57±118.83	9.14E-03
HiQ, *n* = 13	49,504.46±599.28		1968.54±202.07	
Total, *n* = 27	48,467.52±1129.50		2062.93±185.73	
**Selected error-prone 675 loci**				
S200V3, *n* = 14	163.00±15.73	4.13E-01	32.00±3.57	9.32E-03
HiQ, *n* = 13	172.00±13.78		21.54±3.10	
Total, *n* = 27	167.33±15.25		26.96±6.26	
**HLFP, 393 loci**				
S200V3, *n* = 14	81.71±11.47	3.85E-02	25.36±2.31	2.11E-07
HiQ, *n* = 13	90.92±10.44		18.46±2.73	
Total, *n* = 27	86.15±11.75		22.04±4.29	

Two bioinformatics experts manually inspected all of the captured files to evaluate positive calls (Fig 1). Prior to the final evaluation, both experts had inspected more than 100,000 captured images for their read alignment statuses. Each expert determined whether a positive call met the given criteria (Table 2), which was considered to be a false positive, and the others were considered to be true positives. It should be noted that a false positive could have been classified into multiple categories if it satisfied multiple error-prone conditions. Cohen’s kappa coefficient (κ) was 0.6346 for the correlation between the two experts during the first review, and we did not consider in which category a false positive belonged (Fig 1). In order to apply consistent criteria during the second review, the experts discussed patterns of aligned reads with the discordant variants in a consensus meeting, which resulted in Cohen’s kappa coefficient increasing to 0.6513 during the second review. We then classified the calls as follows: (1) a highly likely false positive (HLFP) when both experts agreed it was a false positive, (2) a likely false positive (LFP) when the two experts disagreed, and (3) a likely true positive (LTP) when both experts agreed it was a true positive. This process finally yielded 393 (58.2%) HLFPs, 126 (18.7%) LFPs, and 156 (23.1%) LTPs. The HLFPs were evenly distributed in all of the samples (108.2±11.3, range = 88–133).

**Table 2:** Four distinct error types and their definitions.

Error type	Definition	Example
**Simplicity region**	Sequence called in regions having an unusual composition of nucleotides such as GC/AT-rich and homo-/copolymer sequences.	Fig 2A–2C
**SNV cluster**	Multiple (three or more) variant calls detected in relatively small genomic regions (up to 50 bp).	Fig 2D
**Peripheral sequence read**	Variant calls that occur near to (within 5 bp) of either the start or end of reads.	Fig 2E
**Base inversion**	Sequential calls of 2–3 bp detected with REF-ALT inverted bases.	Fig 2F–2H

**Fig 2:**
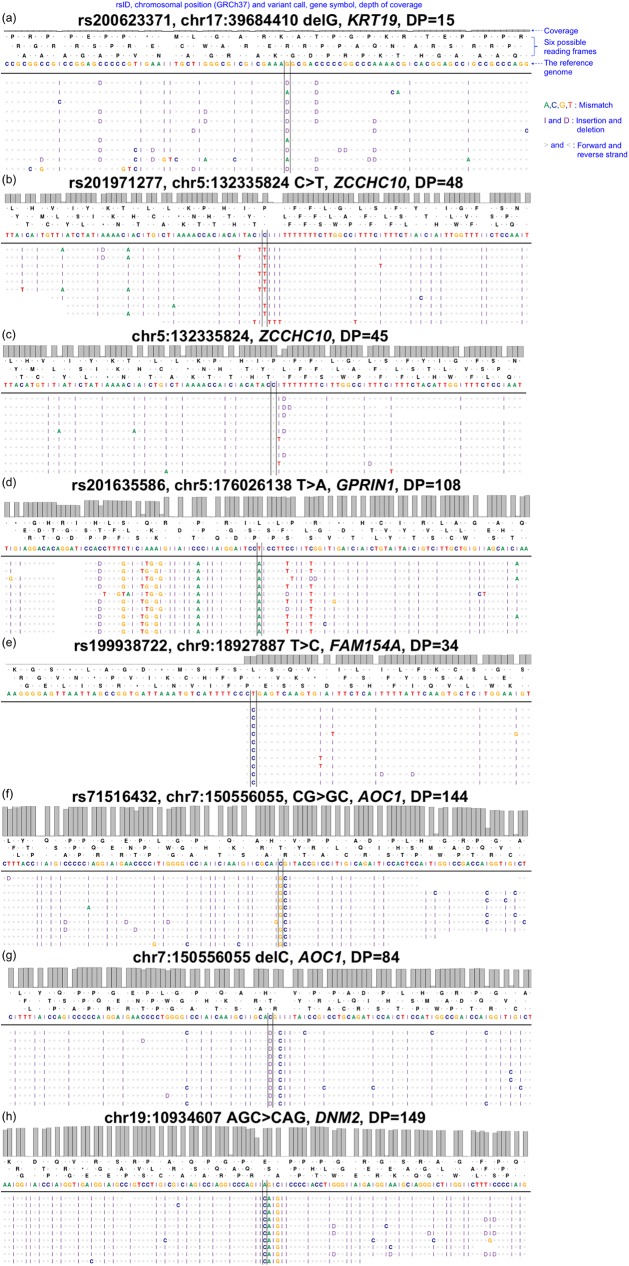
Read alignment patterns and variant calls classified as false positives. We captured the alignment status of reads from BAM files. Each plot shows 10 reads per sample (i.e., not full-depth reads). ‘A,’ ‘C,’ ‘G,’ and ‘T’ indicate mismatch bases against the reference genome. ‘I’ and ‘D’ indicate an insertion and a deletion, respectively, introduced into a read. Left and right angle brackets indicate the direction of aligned reads. (a) A call for rs200623371 is classified as a *simplicity region* error. (b) An erroneous call for rs201971277 immediately after homopolymeric T-repeats was made with HiQ. (c) Reads with T-deletion and T-insertion were made with S200V3 at the same chromosomal position chr5:132335824 as in panel b. (d) An rs201635586 call is classified as an *SNV cluster* error. (e) An rs199938722 call is classified as a *peripheral sequence read*. (f) An example of CG-to-GC inversion for a *base inversion* error, where REF-ALT bases are inverted (chr7:150556054, rs71516432). (g) A misalignment results in erroneous C-deletion and C-insertion calls around a G allele at the same chromosomal position chr7:150556054 as in panel f. (h) An AGC-to-CAG inversion call is shown as an example of a three-base *base inversion*.

**Fig 3:**
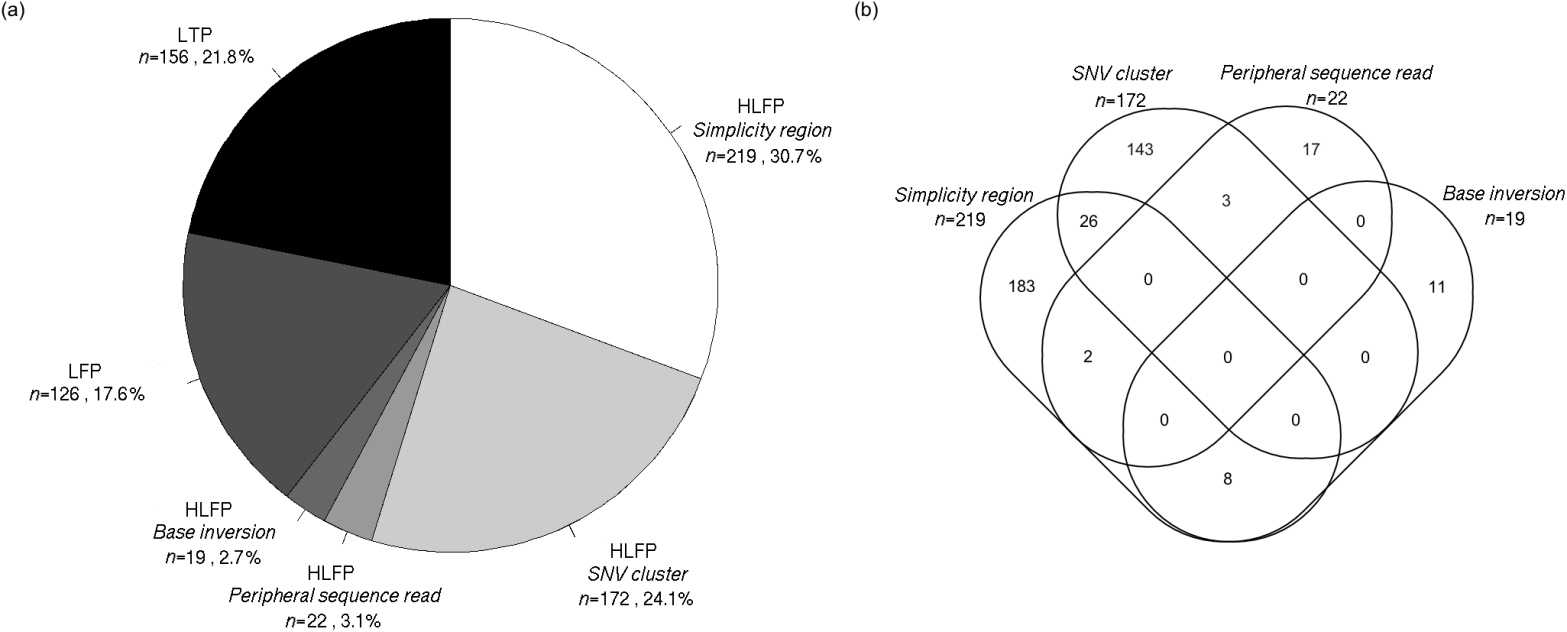
Classification proportions. (a) *Simplicity region* and *SNV cluster* errors dominated, constituting 219 and 172 of the HLFPs, respectively. *Peripheral sequence read* and *base inversion* errors were relatively rare. LFPs and LTPs comprised 126 and 156 of the ultrarare variants, respectively. (b) Of the 393 HLFPs, 354 were distinctly classified into 4 categories, and 39 variants met multiple criteria.

The identified HLFPs were classified into four error types: *simplicity region*, *SNV cluster*, *peripheral sequence read*, and *base inversion* (Table 2). A *simplicity region* represents a sequence variant in a region having unusual compositions of nucleotides such as homo-/copolymer rich and repeat sequences. Fig 2A shows a deletion variant, rs200623371, as an example of a *simplicity region*. Homopolymer length inaccuracies from the Ion Proton sequencer are well known to cause erroneous INDEL calls [15]. This is usually called a ‘homopolymer length error’ but we named it as a *simplicity region* error since not only homopolymer length errors but also many types of alignment biases are present in sequence repeats [16] and low-complexity DNA sequences [17,18]. Of the 393 false positives, 219 (55.7%) were categorized as *simplicity region* errors, hence representing the most common of the four error types (Table 2). Furthermore, 158 (72.1%) of the 219 variants showed WHR values higher than 2.22, which implies that these erroneous variant calls might have been introduced in homopolymer-rich sequences. HiQ is known to handle homopolymers better than S200V3. However, reads from HiQ-based sequencing seem to show confusing signals right after T-repeats, resulting in HLFP calls (Fig 2B). The rs202971277 variant shown in Fig 2B may be an HLFP call due to T-repeats. Though no variant was called from the exomes with S200V3, reads with one or more deletions at the end of the T-repeats were evident (Fig 2C). An *SNV cluster* is another well-known type of variant-calling error where many SNVs are clustered within a relatively small genomic region. Fig 2D shows an example *SNV cluster*: rs201635586. The term ‘SNP cluster’ was introduced by the Genome Analysis Toolkit [14] and is widely used as one of the variant filtering options. Variant calls are classified as an *SNV cluster* if three or more SNVs are detected within a 50-bp region. We found that 172 (43.8%) of the 393 HLFPs were classified as *SNV cluster* (Table 2). A *peripheral sequence read* is a variant called near to the start or the end of a read. Fig 2E shows rs199938722 as an example *peripheral sequence read* detected at the ends of replicated reads. The sequencing quality reportedly decreases toward the end of a read [19–21]. A *base inversion* is an inverted calling pattern for two to three sequential bases. Fig 2F–2H show three *base inversion* calls: (1) a CG-to-GC inversion at chr7:150556054 (Fig 2F), (2) misalignment of the same CG-to-GC inversion reads at the same chromosomal position, chr7:150556054, resulting in erroneous consecutive C-deletion and C-insertion calls around a G allele (Fig 2G), and (3) an AGC-to-CAG inversion call of three bases (Fig 2H). Of the 393 error-prone variants, 22 (6%) and 19 (4.8%) resulted from a *peripheral sequence read* and a *base inversion*, respectively (Table 2 and Fig 3A). Additionally, 39 (9.9%) of the 393 error-prone variants were classified into multiple categories, while the remaining ones satisfied a distinct error-prone condition (Fig 3B).

**Fig 4:**
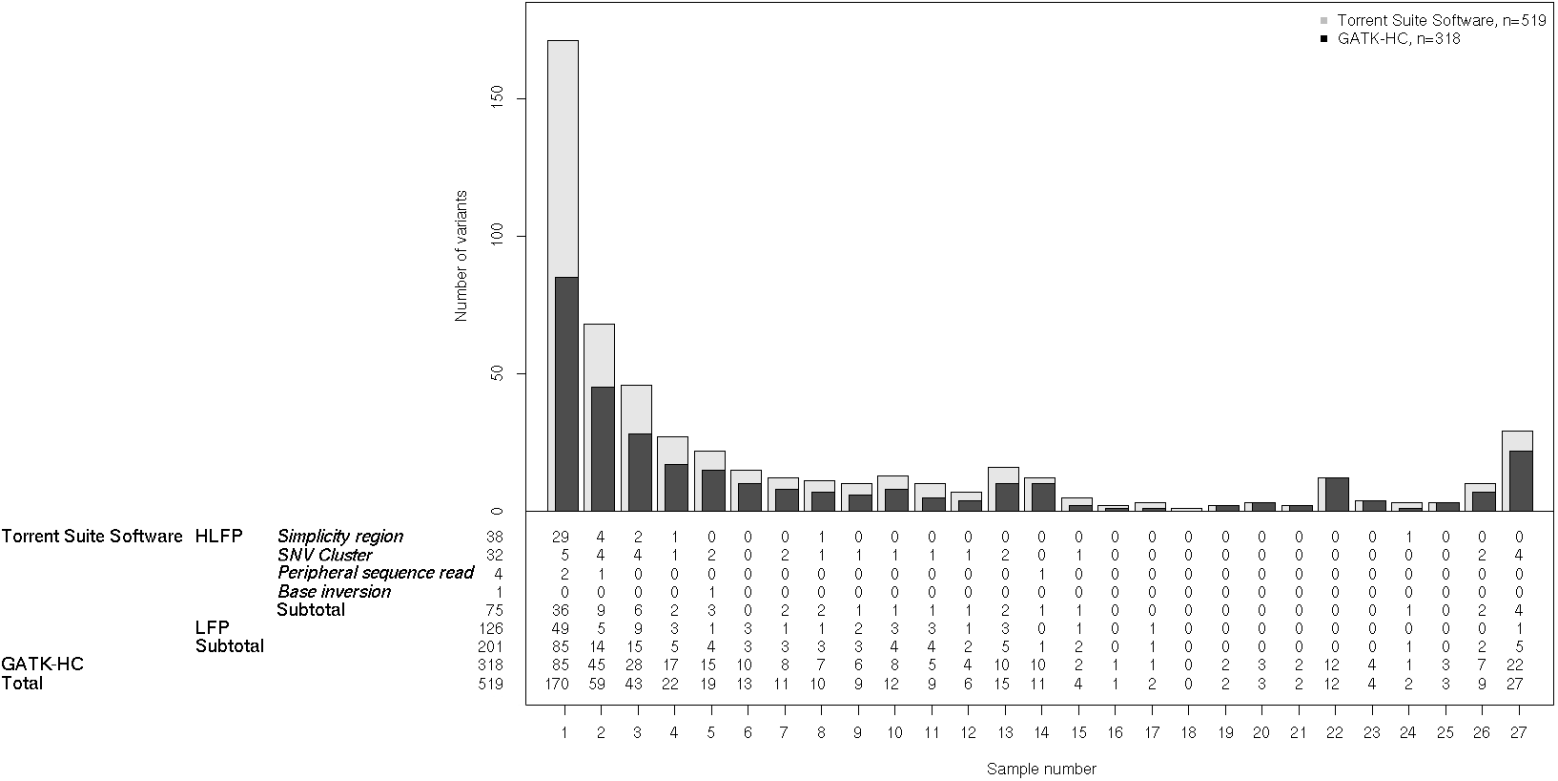
HLFP and LFP variants called by two different algorithms. The variant frequency distribution plot shows the number of variants harbored in certain numbers of samples. Gray and black bars indicate the number of variants called by Torrent Suite Software and the GATK-HC, respectively. Applying local *de novo* assembly technique provided by the GATK-HC corrected 201 out of 519 HLFPs and LFPs to negative calls. Numbers in the panel under the plot indicate the number of variants for each category.

To correct the HLFP and LFP calls, we performed local *de novo* assembly using the GATK-HC for the regions with the 519 variants, including HLFPs and LFPs (Fig 4). We found that 201 (38.7%) variants were not called by the GATK-HC. Among 170 singleton variants that were called by Torrent Suite Software (i.e., those present in only one sample of the cohort), 85 HLFPs and LFPs were discarded after applying the improved algorithm. Five HLFPs and LFPs out of 27 variants found in all of the samples were not reported by the GATK-HC, while 4 of them were *SNV cluster* errors. The number of singletons not called by the GATK-HC vastly outnumbered other variants not called by the GATK-HC that were harbored in multiple samples. Eighty-five (50%) of the 170 HLFPs and LFPs were eliminated by the GATK-HC and were introduced by a single subject.

**Fig 5:**
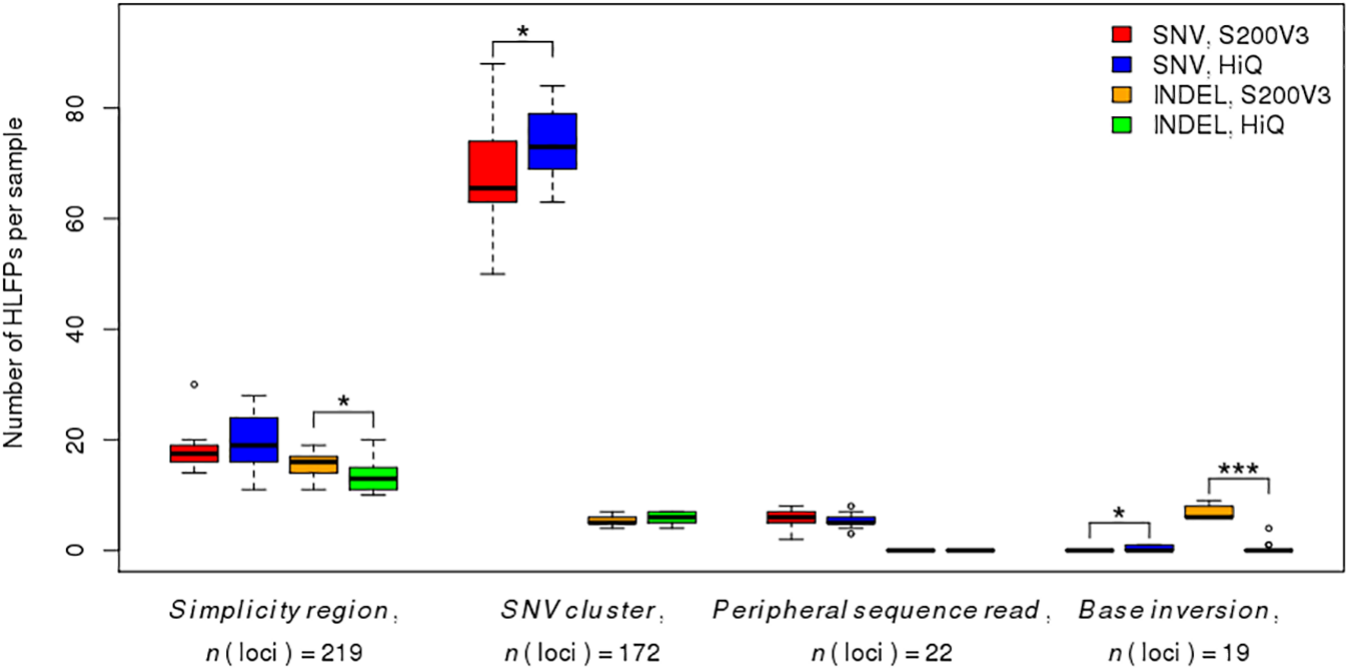
Comparison of sequencing kit-specific effects between S200V3 and HiQ among four variant-calling error types. Box plots of the numbers of HLFP variants per sample in kit groups with SNV and INDEL, respectively. Upper and lower box hinges show the 25th and 75th percentile; whiskers extend from hinges is the maximum and minimum values within 1.5 times the interquartile range (IQR); the median is represented by a horizontal bar in the box. *P < 0.05, ***P < 0.001 by Student’s *t*-test.

For the purpose of analyzing kit-specific effects, 14 and 13 of the 27 subjects were sequenced with S200V3 and HiQ, respectively. All other steps including sequencing devices and analytic software were applied equally. However, we found that 43 (6.4%) of the 675 loci did not show enough read coverages for variant calling (mean depth < 5). Of these 43 loci, 41 (95.3%) and 19 (44.2%) error-prone, poor-coverage loci were found by the S200V3 and HiQ kits, respectively, with 17 (39.5%) error-prone loci found by both kits. A particularly interesting finding was that our algorithm classified 16 (94.1%) of the 17 shared error-prone loci with poor coverage into one of the HLFPs groups: 7 in the simplicity region, 2 peripheral sequence reads, and 7 in the simplicity region and SNV clusters. The remaining single locus was classified as an LFP. As shown in Fig 5, *base inversion* errors differed the most between the two kits. HLFP INDELs of the *base inversion* type were reduced from 12 loci with S200V3 to 3 loci in sequences with HiQ. Additionally, INDELs detected in *simplicity regions* also exhibited a decrease in the error rate (from 65 loci to 51 loci), which supports previous reports of technical improvements in HiQ reducing the INDEL rates [22].

## Discussion

This study focused on ultrarare variants that were positively called in 27 subjects by the Ion Proton sequencer. Among the 675 ultrarare variants, 156 (23.1%) calls were finally classified as LTPs, while the other 519 (76.9%) calls were classified as HLFPs or LFPs. Of the 675 ultrarare variants, 63 were called in all 27 individual samples, which implies that those variants are expected to be artifacts of technical noise in the experiments. It should be noted that these HLFP and LFP variants in the present study have been reported in dbSNP. If researchers find a variant that can be found in public databases, they should carefully consider whether or not this is a rare variant (including private/ultrarare variants) in downstream analyses. Further validation using another sequencing platform and/or chemistry-based method, which was lacking in the present study, may be essential to correctly establish an accurate error model for the HLFP and LFP calls.

The present study proposes a classification scheme for categorizing and hence improving variant-calling algorithms. We first categorized the error patterns into four distinct types: *simplicity region*, *SNV cluster*, *peripheral sequence read*, and *base inversion*. Four distinct error types were obtained heuristically, with *peripheral sequence read* and *base inversion* errors representing new types, while *simplicity region* and *SNV cluster* errors are well-known issues in Next-Generation Sequencing (NGS) data. The *simplicity region* was the dominant error type, and this has been widely known as a specific bias of the Ion Proton sequencer resulting from substantially different sequencing technology that using pH sensor than dye sequencing technique to detect variants [15]. We used the WHR as published online to determine homopolymer-rich sequences, but advanced methods need to be developed to elucidate the characteristics of given sequences, and these characteristics should be considered in the alignment and variant calling process. The *peripheral sequence read* was found by more than one kit and was observed over the entire read length at the ends of reads.

We demonstrated that 38.7% of the erroneous calls were successfully corrected by applying a local *de novo* assembly technique, which requires more computation. This means that the GATK-HC is much better at calling variants than position-based callers that are used in Torrent Suite Software. Moreover, we demonstrated that INDEL error rates for *simplicity region* and *base inversion* were substantially decreased when we used HiQ, which was released more recently [23]. It is suggested that improvements to variant-calling algorithms that consider different error types from the Ion Proton platform with an advanced polymerase will increase the accuracy of next-generation sequencing. We investigated only very limited regions of the whole human exome in the present study, and we were not able to validate the calls since it was not easy to capture the error-prone regions in many cases.

We showed that HiQ is better than S200V3 for INDEL calling. We also tested if the error-prone regions had lower coverage depths than the non-error-prone regions. We defined a non-error-prone region as one where sequence variants were reliably discovered in two public databases (T1GP or ExAC), and compared the coverage depth across different regions: (1) HLFPs (*n* = 393), (2) loci in error-prone regions (*n* = 675), and (3) loci in non-error-prone regions that are reported at least once in T1GP or ExAC (*n* = 70,865). The median depth was significantly lower for error-prone loci than for non-error-prone loci (*P* < 0.05), and tended to be lower for HLFPs than for error-prone loci. The depths when using S200V3 and HiQ were 114.96±80.29 and 123.73±90.77, respectively, for non-error-prone loci, 125.76±122.10 and 133.43±129.51 for error-prone loci, and 114.45±127.20 and 119.67±128.99 for HLFP. It is likely that loci with lower depths of reads tend to result in erroneous calls. Moreover, HiQ chemistry showed significantly improved coverage than S200V3 both in error-prone (*P* < 0.001) and non-error-prone (*P* < 0.001) regions (S1 Fig).

## Supporting information

S1 Fig Comparison of the sequencing kit-specific depth of coverage between S200V3 and HiQ among three variant classes. Box plots of the depth of coverage, with blue, purple, and red colors indicating the variant classes. Hatched and solid patterns indicate S200V3 and HiQ, respectively. ***P < 0.001 by Student’s *t*-test.
(TIF)
