# Supplementary figures and images for: Evaluation of exome variants using the Ion Proton Platform to sequence error-prone regions
Source: PLoS One. 2017 Jul 24;12(7):e0181304. doi: 10.1371/journal.pone.0181304 (PMC5524428; doi:10.1371/journal.pone.0181304)

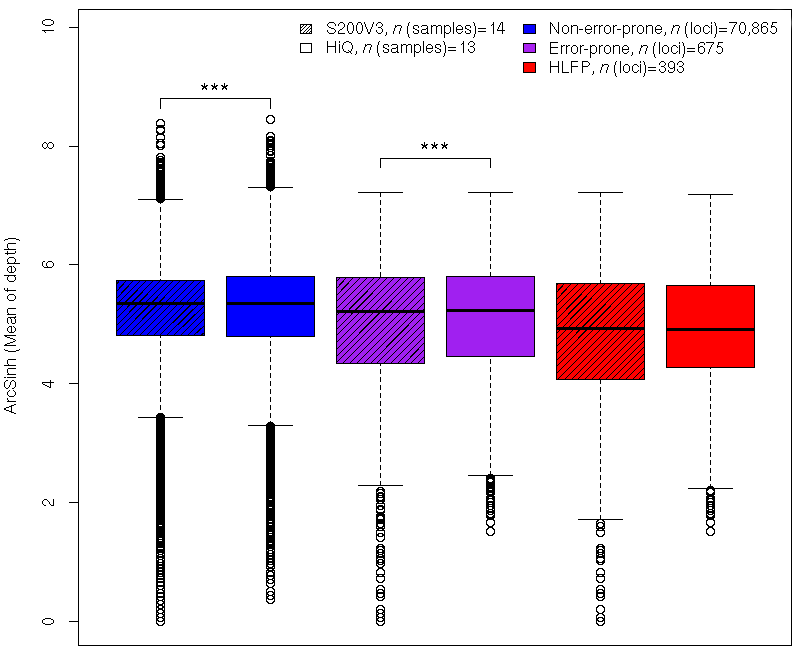

Supplement: S1 Fig — Box plots of the depth of coverage, with blue, purple, and red colors indicating the variant classes. Hatched and solid patterns indicate S200V3 and HiQ, respectively. ***P < 0.001 by Student’s t-test. (TIF) [file pone.0181304.s001.tif]
